# Differential Regulation of Mouse B Cell Development by Transforming Growth Factor β1

**DOI:** 10.1080/1044667031000088057

**Published:** 2002-06

**Authors:** Denise A. Kaminski, John J. Letterio, Peter D. Burrows

**Affiliations:** 1 Department of Microbiology 378 Wallace Tumor Institute The University of Alabama at Birmingham Birmingham AL 35294 USA; 2 Laboratory of Cell Regulation and Carcinogenesis The National Cancer Institute 41 Library Drive Bethesda MD 20892 USA

## Abstract

Transforming growth factor β (TGFβ) can inhibit the *in vitro* proliferation, survival and differentiation of B cell progenitors, mature B lymphocytes and plasma cells. Here we demonstrate unexpected, age-dependent reductions in the bone marrow (BM) B cell progenitors and immature B cells in TGFβ1^-/-^ mice. To evaluate TGFβ responsiveness during normal B lineage development, cells were cultured in interleukin 7 (IL7)±TGFβ. Picomolar doses of TGFβ1 reduced pro-B cell recoveries at every timepoint. By contrast, the pre-B cells were initially reduced in number, but subsequently increased compared to IL7 alone, resulting in a 4-fold increase in the growth rate for the pre-B cell population. Analysis of purified BM sub-populations indicated that pro-B cells and the earliest BP1^-^ pre-B cells were sensitive to the inhibitory effects of TGFβ1. However, the large BP1^+^ pre-B cells, although initially reduced, were increased in number at days 5 and 7 of culture. These results indicate that TGFβ1 is important for normal B cell development *in vivo*, and that B cell progenitors are differentially affected by the cytokine according to their stage of differentiation.


*Developmental Immunology*, 2002 Vol. 9 (2), pp. 85–95

Taylor & Francis healthsciences

### Differential Regulation of Mouse B Cell Development by Transforming Growth Factor β1

DENISE A. KAMINSKI[a,*], JOHN J. LETTERIO[b] and PETER D. BURROWS[a,†]

[a]*Department of Microbiology, 378 Wallace Tumor Institute, The University of Alabama at Birmingham, Birmingham, AL 35294, USA;* [b]*Laboratory of Cell Regulation and Carcinogenesis, The National Cancer Institute, 41 Library Drive, Bethesda, MD 20892, USA*

*(Revised 4 January 2003)*

Transforming growth factor β (TGFβ) can inhibit the *in vitro* proliferation, survival and differentiation of B cell progenitors, mature B lymphocytes and plasma cells. Here we demonstrate unexpected, age-dependent reductions in the bone marrow (BM) B cell progenitors and immature B cells in TGFβ$1^{-/-}$ mice. To evaluate TGFβ responsiveness during normal B lineage development, cells were cultured in interleukin 7 (IL7) ± TGFβ. Picomolar doses of TGFβ1 reduced pro-B cell recoveries at every timepoint. By contrast, the pre-B cells were initially reduced in number, but subsequently increased compared to IL7 alone, resulting in a 4-fold increase in the growth rate for the pre-B cell population. Analysis of purified BM sub-populations indicated that pro-B cells and the earliest $BP1^-$ pre-B cells were sensitive to the inhibitory effects of TGFβ1. However, the large $BP1^+$ pre-B cells, although initially reduced, were increased in number at days 5 and 7 of culture. These results indicate that TGFβ1 is important for normal B cell development *in vivo*, and that B cell progenitors are differentially affected by the cytokine according to their stage of differentiation.

*Keywords*: B cell progenitor; Bone marrow; IL7; Pre-B cell; Pro-B cell; TGFβ

*Abbreviations*: BM, bone marrow; HC, antibody heavy chain; BCP, B cell progenitor; TGFβ, transforming growth factor β; IL7, interleukin 7; LM, littermate

### INTRODUCTION

Transforming growth factor β (TGFβ) is distinguished among cytokines in its involvement in multiple biological processes, eliciting unique responses according to context (Massague *et al.*, 1992; Rifkin *et al.*, 1993; McCartney-Francis and Wahl, 1994; Bottinger *et al.*, 1997). Its overlapping functions include regulation of embryogenesis (Dickson *et al.*, 1995; Kaartinen *et al.*, 1995; Bonyadi *et al.*, 1997; Sanford *et al.*, 1997), cell cycle and viability (Ravitz and Wenner, 1997; Hocevar and Howe, 1998) and cellular adhesion (Roberts *et al.*, 1992; Wahl, 1994; Kim and Yamada, 1997; Letterio and Roberts, 1998). The interplay of these TGFβ-regulated processes controls the development and function of the immune system (Yaswen *et al.*, 1996; Letterio and Roberts, 1998; Larsson *et al.*, 2001).

Limitations to studying TGFβ effects *in vivo* are imposed by its importance during embryogenesis. Gene-targeting of each isoform (TGFβ1, 2 or 3) as well as each of the two receptor subunits (TβRI and TβRII) results in lethality at or prior to birth (Dickson *et al.*, 1995; Kaartinen *et al.*, 1995; Martin *et al.*, 1995; Bonyadi *et al.*, 1997; Sanford *et al.*, 1997). The earliest lethality is seen in $T\beta RI^{-/-}$, $T\beta RII^{-/-}$ (Larsson *et al.*, 2001), and in ~50% of TGFβ$1^{-/-}$ embryos (Shull *et al.*, 1992; Kulkarni *et al.*, 1993), which expire at ~10.5 days post-coitus, due to aberrantly developed yolk sac vasculature and anemia. The embryonic anemia *in vivo* is likely a secondary result of inadequate vascularization, since endothelia from the TGFβ1 mutant embryos fail to differentiate in culture (Martin *et al.*, 1995), whereas *in vitro* development of yolk sac-derived $T\beta RI^{-/-}$ hematopoietic progenitors into various blood cell lineages is similar to controls (Larsson *et al.*, 2001).

The importance of TGFβ in immune regulation is underscored by the phenotype of TGFβ$1^{-/-}$ mice, which have multiple abnormalities, including systemic inflammation to which they succumb by 3–5 weeks after birth

*Present Address: Department of Molecular Genetics and Microbiology, University of Massachusetts Medical School, 55 Lake Avenue, Worcester, MA 01655, USA.

†Corresponding author. Tel.: +1-205-934-6529. Fax: +1-205-934-1875. E-mail: peterb@uab.edu

ISSN 1044-6672 print © 2002 Taylor & Francis Ltd
DOI: 10.1080/1044667031000088057

(Shull *et al.*, 1992; Kulkarni and Karlsson, 1993; Kulkarni *et al.*, 1993; Diebold *et al.*, 1995; Kulkarni *et al.*, 1995; McCartney-Francis *et al.*, 1997). The inflammatory disease of TGFβ1$^{-/-}$ mice is attenuated when T cells are eliminated, implicating T cells as a major mediator of inflammation (Kulkarni and Karlsson, 1993; Diebold *et al.*, 1995; Kulkarni *et al.*, 1995; Borkowski *et al.*, 1996; Letterio *et al.*, 1996; McCartney-Francis *et al.*, 1997; Nakabayashi *et al.*, 1997; Kobayashi *et al.*, 1999; McLennan *et al.*, 2000).

Once a hematopoietic progenitor enters the B lineage pathway, it progresses through a number of developmental stages defined by expression of cell surface differentiation antigens (Hardy *et al.*, 1991; Rolink *et al.*, 1999), cell cycle status (Osmond, 1991; Itoh *et al.*, 1996), antibody variable region gene rearrangements (Hardy *et al.*, 1991; Hardy, 1992; Li *et al.*, 1993; Papavasiliou *et al.*, 1997; Rolink *et al.*, 1999), responsiveness to and requirements for interleukin 7 (IL7) receptor signaling (Peschon *et al.*, 1994; Candeias *et al.*, 1997; Marshall *et al.*, 1998) and interaction with the bone marrow (BM) stroma (King *et al.*, 1988; Gimble *et al.*, 1989; Dittel *et al.*, 1993; Dittel and LeBien, 1995; Borghesi *et al.*, 1997). The IL7 receptor is indispensable for mouse B cell development during the V-to-DJ heavy chain (HC) variable region gene rearrangement process (Corcoran *et al.*, 1998). Acquisition of μHC and formation of the pre-B cell receptor are associated with a decreased IL7 dose-response threshold (Marshall *et al.*, 1998). The resulting increases in IL7 sensitivity may be responsible for the large size and mitotic status of early/intermediate pre-B cells. Late pre-B cells exit the cell cycle and undergo light chain V–J rearrangement in preparation for full antibody assembly and surface expression on the more differentiated B cell (Meffre *et al.*, 2000; Melchers *et al.*, 2000).

The effects of exogenous TGFβ have been examined in cultured B lineage cells representative of almost every developmental stage, and are usually inhibitory. Early studies showed that TGFβ inhibits the proliferative response of BM B cell progenitors (BCP) to IL7, and that it can inhibit κLC acquisition in a differentiating B lineage cell line (Lee *et al.*, 1987; Kincade *et al.*, 1989). Similar observations have been made for κ light chains in human fetal BM cultures (Rehmann and LeBien, 1994). However, these studies did not distinguish the effects of TGFβ on pro-B versus pre-B cells within one system. Induction of the transcriptional regulator Id3 by TGFβ, together with inhibition of cell cycling and *Rag1* mRNA expression has also been demonstrated (Kee *et al.*, 2001). TGFβ effects at later mature B and plasma cells stages are almost exclusively negative with the exception of inducing IgA isotype switching (Kim and Kagnoff, 1990; Lebman *et al.*, 1990; Shockett and Stavnezer, 1991). These studies indicate that TGFβ can inhibit the *in vitro* survival, proliferation and differentiation of antibody-producing B cells at all stages of development.

An inhibitory role for TGFβ in the immune system is supported by the phenotype of juvenile TGFβ1$^{-/-}$ mice (Christ *et al.*, 1994). Infiltrates of plasma cells are found in secondary lymphoid organs and also in non-lymphoid tissues where they accompany inflammatory infiltrates of other hematopoietic cells (Christ *et al.*, 1994; Kulkarni *et al.*, 1995; van Ginkel *et al.*, 1999). The mice also have increased levels of anti-nuclear and anti-collagen serum antibodies (Dang *et al.*, 1995; Yaswen *et al.*, 1996) and hyperproliferation in the splenic B cell follicles (Christ *et al.*, 1994).

These observations, together with the described inhibitory effects of TGFβ on *in vitro* B cell development, predicted the expansion of B cell progenitors in the absence of TGFβ *in vivo*. We found instead an age-related deficiency in B cell development in TGFβ1$^{-/-}$ mice. The complication of the co-existing inflammatory disease in these mice lead us to re-examine the *in vitro* effects of TGFβ1 on defined sub-populations of normal BM B lineage cells. The results of this combined approach indicate that deficiencies in the earliest B cell progenitors in the TGFβ1$^{-/-}$ mice are likely to be due to secondary effects of the phenotype, since pro-B cell growth is inhibited by TGFβ1 *in vitro*. By contrast, TGFβ1 increases the recovery of the large pre-B cells. Collectively, these observations demonstrate that TGFβ1 is required for normal B cell development *in vivo*, and indicate differential sensitivity of B cell progenitors to TGFβ according to their stage of differentiation.

### MATERIALS AND METHODS

### Flow Cytometry

TGFβ1$^{-/-}$ mice were derived from TGFβ1$^{+/-}$ crosses as described (Kulkarni *et al.*, 1993). Erythrocyte-depleted BM cells were recovered from TGFβ1$^{-/-}$ mice and aged-matched TGFβ1$^{+/+}$ littermate controls on a mixed C57BL/6J × SVJ/129 background. All incubations for flow cytometry were on ice for 15 min, followed by washing with 1% FBS in PBS. Aliquots of $10^6$ cells from each mouse were stained with combinations of fluorochrome-conjugated antibodies specific for CD43 (S7), BP1 (6C3/BP1), HSA (M1/69), Mac1 (M1/70), Thy1.2 (30H12) and B220 (RA3-6B2) from BD Pharmingen (San Diego, CA), IgD (SBA-1) and goat anti-mouse IgM from Southern Biotechnology Associates (Birmingham, AL). Stained cells were analyzed using a Becton-Dickinson FACSCalibur flow cytometer (San Diego, CA). Values from TGFβ1$^{+/+}$ and TGFβ1$^{-/-}$ mice were compared using a Student's *t*-Test.

### Cell Culture

Erythrocyte-depleted BM from 4- to 5-week-old female C57BL/6 mice was purified by centrifugation over a Lympholyte M gradient (Cedar Lane, Hornby, Ont., Canada). B220$^{+}$ cells were isolated by positive selection with magnetic beads (Miltenyi, Auburn, CA); sorting efficiency was assessed by flow cytometry to be 85 ± 8%. FACS®-sorted cells were purified with a MoFlo flow cytometer (Cytomation, Fort Collins, CO) using anti-CD19 (clone 6D5 from Southern Biotechnology Associates, Birmingham, AL), anti-BP1 and anti-IgM (as above). $5 \times 10^4$ sorted cells/ml were plated in complete IMDM (5% FCS, $5 \times 10^{-5}$ M 2ME, 1% each L-glutamine, penicillin/streptomycin, non-essential amino acids) and treated with 10 ng/ml recombinant mouse IL7 (PeproTech, Rocky Hill, NJ) ± recombinant human TGFβ1 (R and D Systems, Minneapolis, MN). Two doses of 0.04 ng/ml (1.6 pM) or 1 ng/ml (40 pM) were compared in all of the experiments shown here because dose-response experiments showed distinct read-outs at these two concentrations. Harvested cells were counted by Trypan Blue exclusion and surface-stained for B220 (as above) and sIgM [goat anti-mouse IgM$^{cy5}$ from Jackson Laboratories (Bar Harbor, ME) or MB86$^{Alexa647}$ from John Kearney (Birmingham, AL)], or control antibodies. These cells were fixed in 1% paraformaldehyde, permeablized with Tween 20 and stained intracellularly with a goat anti-mouse $\mu^F$ antibody. For some experiments, the CytoFix/Cytoperm kit from Pharmingen was used according to the manufacturer's instructions. The pre-B cell growth rate was calculated as (pre-B cell number recovered on day 7 − pre-B cell number recovered on day 3)/4 days = cells/day.

### RESULTS

### Age-dependent BM B Lineage Cell Reductions in TGFβ1$^{-/-}$ Mice

Flow cytometry was used to examine the proportions (Fig. 1A,B) and absolute numbers (Fig. 1C) of B220$^+$ B lineage cells in the BM of neonatal (1.5-week-old) and juvenile (3.5-week-old) TGFβ1$^{-/-}$ mice. The 1.5 week-old TGFβ1$^{-/-}$ mice were comparable to the TGFβ1$^{+/+}$ littermate (LM) controls at the early, sIgM$^-$ and later, sIgM$^+$ stages. By contrast, 3.5-week-old mice showed a significant 2.6-fold reduction in the percentage of B220$^+$sIgM$^-$ cells, corresponding to a significant 4.6-fold reduction in absolute cell number. Absolute numbers and percentages of B220$^+$sIgM$^+$ B cells were also reduced, although not consistently.

The cell surface marker system described by Hardy *et al.* (1991) was used to further define the B lineage developmental stages affected by the TGFβ1 deficiency, and the results were calculated both as a percentage of total BM (Fig. 2A,B) and as absolute cell numbers (Fig. 2C). In TGFβ1$^{-/-}$ mice examined at 3.5 weeks of age, the percentage of cells in Fraction B (B220$^+$CD43$^+$HSA$^+$BP1$^-$), including pro- and pre-B cells (Wasserman *et al.*, 1997), was not significantly changed, although the absolute numbers of these cells were 2.6-fold reduced. The percentage and absolute number of pre-B cells in Fraction C (B220$^+$CD43$^+$HSA$^+$BP1$^+$)

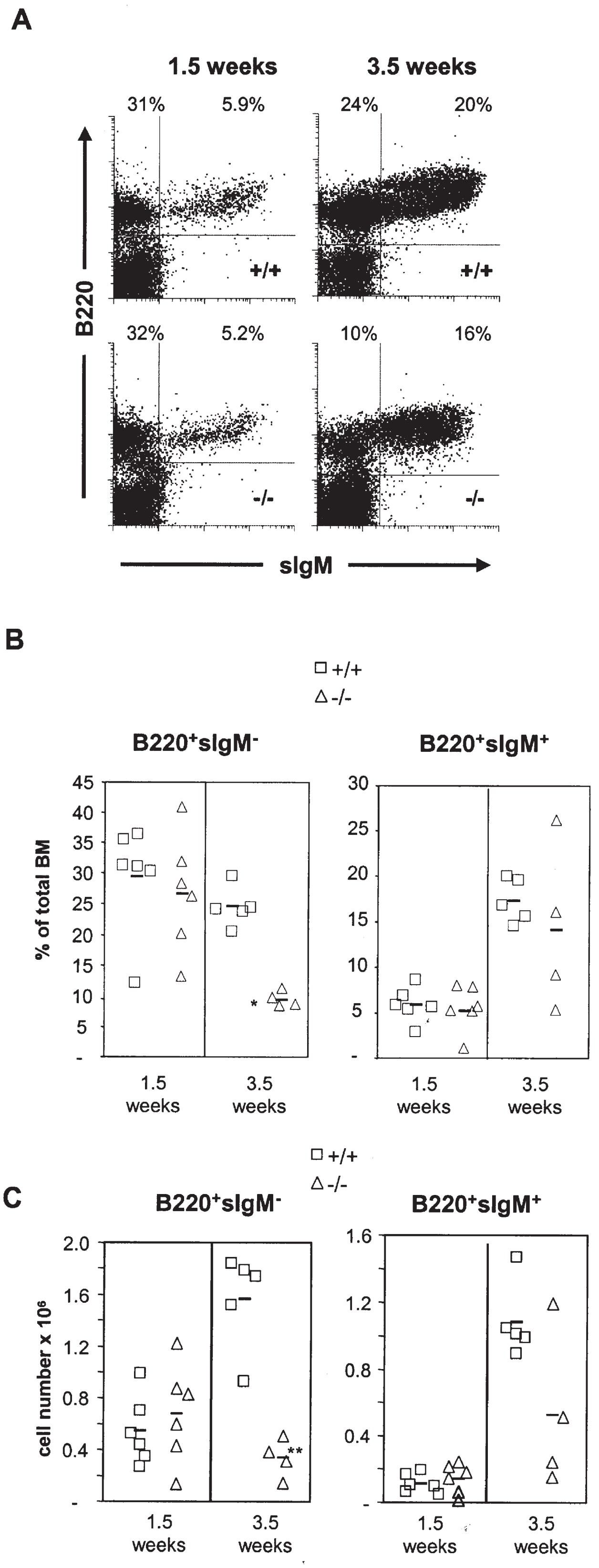

FIGURE 1 B cell development in TGFβ1$^{-/-}$ mice. BM from TGFβ1$^{-/-}$ mice and age-matched TGFβ1$^{+/+}$ (LM) controls was prepared for flow cytometry as indicated in "Methods" section. (A) Profiles of gated lymphocytes showing expression of B220 and sIgM. Values indicated are the per cent of total BM. (B) Percentage of total BM for individual mice. $*p = 0.0001$ for the B220$^+$sIgM$^-$ BCP between TGFβ1$^{-/-}$ and LM controls. (C) Absolute numbers of cells for the populations shown in A and B. $**p = 0.008$.

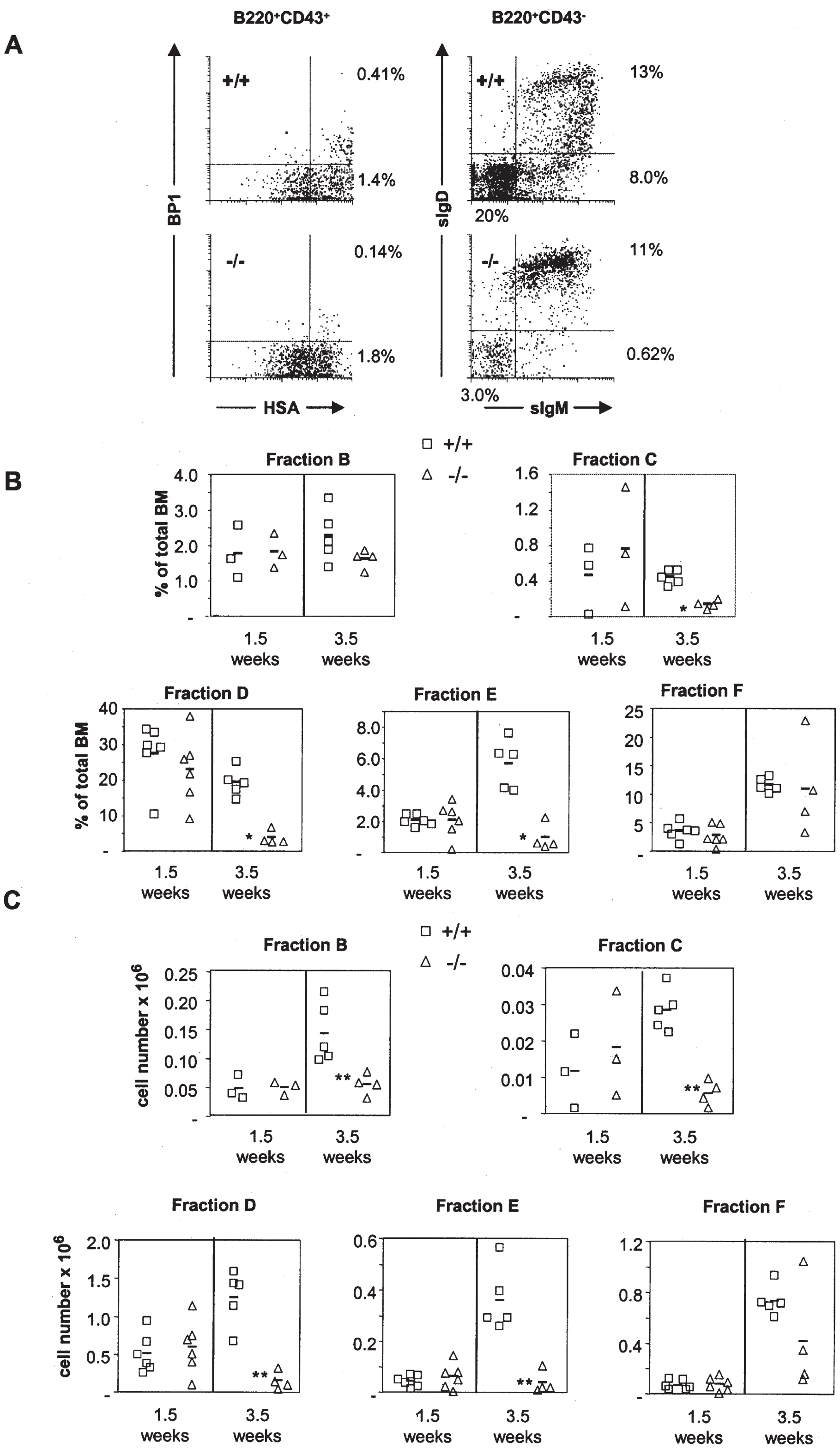

FIGURE 2 B cell progenitor deficiencies in juvenile TGFβ1$^{-/-}$ BM. BM lymphocytes from 3.5-week-old TGFβ1$^{-/-}$ mice and LM controls were prepared for flow cytometry as in Fig. 1 using the indicated markers. (A) Representative flow cytometry profiles gated on the B220$^{+}$CD43$^{+}$ or B220$^{+}$CD43$^{-}$ lymphocyte populations as indicated. Values indicated are the per cent of total BM. (B) Percentage of total BM for individual mice. *$p \leq 0.001$ between TGFβ1$^{-/-}$ and LM controls. (C) Absolute numbers of B lineage cells for mice represented in A and B. **$p \leq 0.02$ between TGFβ1$^{-/-}$ and LM controls.

TABLE I Frequency* of BM B lineage compartment reductions in TGFβ1$^{-/-}$ mice

| | B220$^+$ | | B Lineage fraction† | | | | |
|---|---|---|---|---|---|---|---|
| Age | sIgM$^-$ | sIgM$^+$ | B | C | D | E | F |
| 1–2 weeks | 1/7 | 1/7 | 0/4 | 0/4 | 1/7 | 1/7 | 3/7 |
| >2 weeks | 10/14 | 3/14 | 7/11 | 10/13 | 11/12 | 9/12 | 7/12 |

* Number of TGFβ1$^{-/-}$ mice with reductions in absolute cell numbers of ≥2-fold compared to LM controls per number of TGFβ1$^{-/-}$ mice examined.
† According to (Hardy *et al.*, 1991; Li *et al.*, 1993; Li *et al.*, 1996).

were significantly reduced by 3.0- and 5.0-fold, respectively. The late pre-B cells in Fraction D (B220$^+$CD43$^-$IgM$^-$IgD$^-$) were proportionally reduced by 4.6-fold and in absolute cell number by 8.2-fold. The subsequent immature B cells, Fraction E (B220$^+$CD43$^-$IgM$^+$IgD$^-$) were proportionally reduced by 5.7-fold and in absolute number by 9.2-fold. Mature B cells (B220$^+$CD43$^-$IgM$^+$IgD$^+$, Fraction F) in the BM were variably increased or decreased in proportion and in absolute number (Fig. 2C) as was observed in the periphery [not shown and Christ *et al.* (1994)]. It should be noted that Fraction A (B220$^+$CD43$^+$HSA$^-$BP1$^-$) is not consistently affected in the TGFβ1$^{-/-}$ mice (Fig. 2A and not shown); however, not all the cells in this population are progenitors of the B lineage (Tudor *et al.*, 2000).

*In vivo*, the reductions seen in Fraction B were statistically significant when calculated as absolute numbers of cells recovered (2.6-fold), but not as a percentage of total BM cells. This might suggest that Fraction B itself is unchanged, and that the smaller size of the TGFβ1$^{-/-}$ mice (Shull *et al.*, 1992; Boivin *et al.*, 1995; Kulkarni *et al.*, 1995), and therefore smaller bone cavity, is the cause of this reduction in cell number. However, normalizing the cell number to body weight of each mouse still results in a significant reduction in Fraction B, although the degree of reduction is less, at 1.8-fold (+/+, $1.46 \times 10^4 \pm 4.98 \times 10^3$ cells/g; −/−, $8.08 \times 10^3 \pm 2.27 \times 10^3$ cells/g, $p = 0.048$). The reduction in Fraction C, however, is significant regardless of how the data are calculated: as a proportion of total BM (3.0-fold), as an absolute cell number (5.0-fold), and also as a normalized cell number [3.6-fold (+/+, $2.90 \times 10^3 \pm 632$ cells/g; −/−, $810 \pm 463$ cells/g, $p = 0.00091$)].

A summary of a more extensive analysis of mice at different ages is shown in Table I as the frequency of TGFβ1$^{-/-}$ mice with reductions of ≥2-fold in the absolute numbers of BM B lineage cells. These mice are not included in Fig. 2 because a different number of bones per mouse were used for the analysis. Although there is some variability, these results confirm the age-dependent decrease in BCP in the TGFβ1$^{-/-}$ mice. The frequencies of mature B cells are reduced in slightly more than half of the mice, which may be due to variable experiences of these recirculating cells in the periphery. Phenotypic variability in TGFβ1$^{-/-}$ mice has been noted in other contexts, for example, inflammation in organs other than heart and lung (Shull *et al.*, 1992; Kulkarni and Karlsson, 1993; Boivin *et al.*, 1995; Kulkarni *et al.*, 1995).

Activated T cells are responsible for much of the characteristic inflammatory phenotype of the TGFβ1$^{-/-}$ mouse (Diebold *et al.*, 1995; Kobayashi *et al.*, 1999). To ask whether the BCP reductions in the TGFβ1$^{-/-}$ mice were associated with an altered BM microenvironment, Thy1.2 and Mac1 were used as markers for BM T and myeloid cells, respectively. Although the Thy1.2$^+$ population was proportionally increased in all 3.5-week-old mice examined (Fig. 3A), this did not correlate with increased cell numbers in most mice (Fig. 3B). The proportions of Mac1$^+$ cells were increased in half of the mice examined (Fig. 3C); however, once again, this generally did not correspond to an increase in the absolute numbers of myeloid lineage cells (Fig. 3D). One explanation for the observed discrepancy between the percentages and absolute cell numbers may again be the smaller size of the TGFβ1$^{-/-}$ mice (Shull *et al.*, 1992; Boivin *et al.*, 1995; Kulkarni *et al.*, 1995). However, when the data were normalized to body weight, a similar pattern was observed for Thy1.2 (+/+, $1.8 \times 10^4 \pm 1.1 \times 10^4$ cells/g; −/−, $4.4 \times 10^4 \pm 2.6 \times 10^4$ cells/g), and for Mac1 (+/+, $7.5 \times 10^4 \pm 1.1 \times 10^4$ cells/g; −/−, $11.1 \times 10^4 \pm 4.8 \times 10^4$ cells/g). These findings show that there is an altered cellular composition of the BM in the TGFβ1$^{-/-}$ mice, including changes in the proportions of

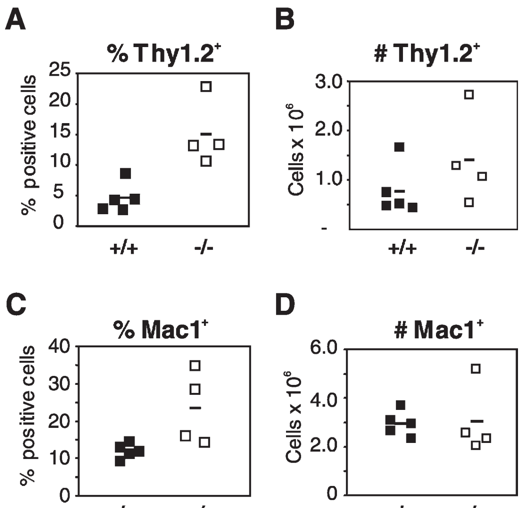

FIGURE 3 T and myeloid cell compartments of TGFβ1$^{-/-}$ BM. Flow cytometry analysis of 3.5-week-old TGFβ1$^{-/-}$ mice and controls was performed using the markers indicated. Proportions (A,C) and absolute numbers (B,D) of bone marrow cells expressing T (Thy1.2; A,B) and myeloid (Mac1; C,D) lineage markers are shown. The Thy 1.2$^+$ cells are within the lymphoid gate. $p > 0.05$ for all sample sets.

myeloid and T lineage cells. However, the absolute numbers of these cells are not consistently increased in all of the mice that had an equally severe reduction in BCP. Therefore, a global disruption of the BM microenvironment seems unlikely, although our analysis does not exclude possible inhibitory effects of inflammatory/myelopoietic foci on the B lineage cells in TGFβ1$^{-/-}$ BM.

### Exogenous TGFβ1 Effects upon Normal Pro- and Pre-B Cells *In Vitro*

The complex pathology of the TGFβ1$^{-/-}$ mice and the lack of correlation between the frequency of Thy1.2$^{+}$ and Mac1$^{+}$ BM cells and the BCP deficiency lead us to re-examine the effects of TGFβ1 on B cell development *in vitro*. We asked whether TGFβ1 might be beneficial for B cell development as suggested by the phenotype of the TGFβ1$^{-/-}$ mice. B220$^{+}$ BM B lineage cells from normal (C57BL/6) mice were treated with TGFβ1 in the presence of IL7, a cytokine that stimulates BCP proliferation prior to the late pre-B cell stage (Hardy *et al.*, 1991; Marshall *et al.*, 1998). Stromal cells were not included in our system due to their ability to produce and respond to TGFβ (Dittel *et al.*, 1993; Dittel and LeBien, 1995; Robledo *et al.*, 1998; Olsen *et al.*, 2001).

Intracellular (i.c.) μHC expression in B220$^{+}$sIgM$^{-}$ BCP was used to identify pre-B cells recovered from cultures of IL7-stimulated B220$^{+}$ BM cells (Fig. 4A). At day 3 in the control sample of IL7 alone (first column), there was a predominance of pre-B cells; however, the pro-B cell-enriched population (i.c. μHC$^{-}$ BCP) predominated by day 7. It should be noted that a majority of the pre-B cells in the starting population are late pre-B cells, which do not proliferate in response to IL7 (Hardy *et al.*, 1991; Marshall *et al.*, 1998). During the course of a week-long culture, these cells should either expire or mature to become sIgM$^{+}$ B cells and thus be excluded from the analysis. Meanwhile, IL7-responsive pro-B cells accumulate and predominate in the cultures by day 7.

Addition at day 0 of either 0.04 or 1 ng/ml TGFβ1 to the IL7 cultures resulted in little change at day 3 in the percentage of pre-B cells compared to IL7 alone (Fig. 4A). However, both treatments resulted in a consistent reduction in the total viable cell numbers recovered (not shown) corresponding to a 2-fold reduction in both pro- and pre-B cell numbers at day 3 of culture [Fig. 4B (*Bottom*) and C, respectively].

At the later timepoints of 5 and 7 days, TGFβ1 treatment resulted in a small increase in the proportion of pre-B cells in comparison to IL7 alone (Fig. 4A). This was partially due to reductions in pro-B cell numbers in the presence of TGFβ1 (Fig. 4B). In contrast to reductions in pre-B cell numbers seen at day 3 of culture, treatment with 0.04 ng/ml TGFβ1 resulted in a modest increase in the numbers of pre-B cells recovered at 5 and 7 days. By contrast, treatment with 1 ng/ml TGFβ1 showed minor pre-B cell reductions at later timepoints (Fig. 4C).

The initial reductions in pre-B cells indicate that some cells are likely to be sensitive to the inhibitory effects of TGFβ1. However, the remaining pre-B cell population either has, or acquires a very high proliferative capacity, indicated by a significant 4-fold increase in the rate of growth between 3 and 7 days (Fig. 4D).

To identify the populations responsible for these distinct outcomes, we initiated similar experiments using sort-purified BM. The BP1 cell surface marker was used to subdivide normal BM into two sub-populations of CD19$^{+}$sIgM$^{-}$ BCP: (1) BP1$^{-}$ cells, ~60% of which are i.c. μHC$^{+}$ and (2) large BP1$^{+}$ cells, ~90–99% i.c. μHC$^{+}$ (not shown). Between days 3 and 7 in culture with IL7 alone, the number of BP1$^{-}$-derived pro- and pre-B cells increased (Fig. 5A,B, respectively); in each case and at all time points, 0.04 and 1 ng/ml exogenous TGFβ1 resulted in reduced cell recovery. TGFβ1-mediated reductions in pro-B cell recoveries were also confirmed using CD19$^{+}$ Rag1$^{-/-}$ BM as a source of pro-B cells (not shown). In contrast to the cell growth observed with IL7 alone in cultures of BP1$^{-}$ BCP, the large BP1$^{+}$ pre-B cells decreased in number between days 3 and 7 (Fig. 5C). In this context, treatment with either dose of TGFβ1 resulted in an initial decrease in pre-B cell recoveries, but subsequently, low-dose TGFβ1 treatment resulted in a net increase at days 5 and 7 of culture (2.1 ± 0.7 and 3.0 ± 0.6-fold increase, respectively over IL7 alone; $n = 3$ experiments).

Figure 5D summarizes the effects of low-dose TGFβ1 treatment where the diameter of each circle relative to the control (1.0) represents the average fold change in cell number. As seen on day 3, each stage analyzed contains cells that are sensitive to the inhibitory effects of TGFβ1. TGFβ1-mediated reductions continue over time for BP1$^{-}$ BCP-derived pro- and pre-B cells, whereas the BP1$^{+}$ fraction contains cells that are positively affected by exogenous TGFβ1 treatment at a low-dose (0.04 ng/ml). At a later timepoint in the culture, IgM$^{+}$ B cells accumulate as well (Fig. 5D and data not shown).

### DISCUSSION

We have demonstrated an age-dependent reduction in BM B lineage cells in TGFβ1$^{-/-}$ mice. The deficiency is apparent as early as Hardy's Fraction B, containing pro-B cells and extends through the immature B cell stage. The mice also have variable increases in the proportions and numbers of BM Thy1.2$^{+}$ and Mac1$^{+}$ cells, but these do not consistently correlate with the reduction in B lineage cells. Subsequent studies of the *in vitro* effects of exogenous TGFβ1 on BM B lineage cells from normal mice showed reductions in pro-B cell recoveries as early as day 3 and continuing through day 7 of incubation at pg/ml doses of TGFβ1. Although the same cultures showed an initial decrease in pre-B cell numbers, this was followed by increases at days 5 and 7 translating into a 4-fold increase in the rate of growth for the pre-B cell

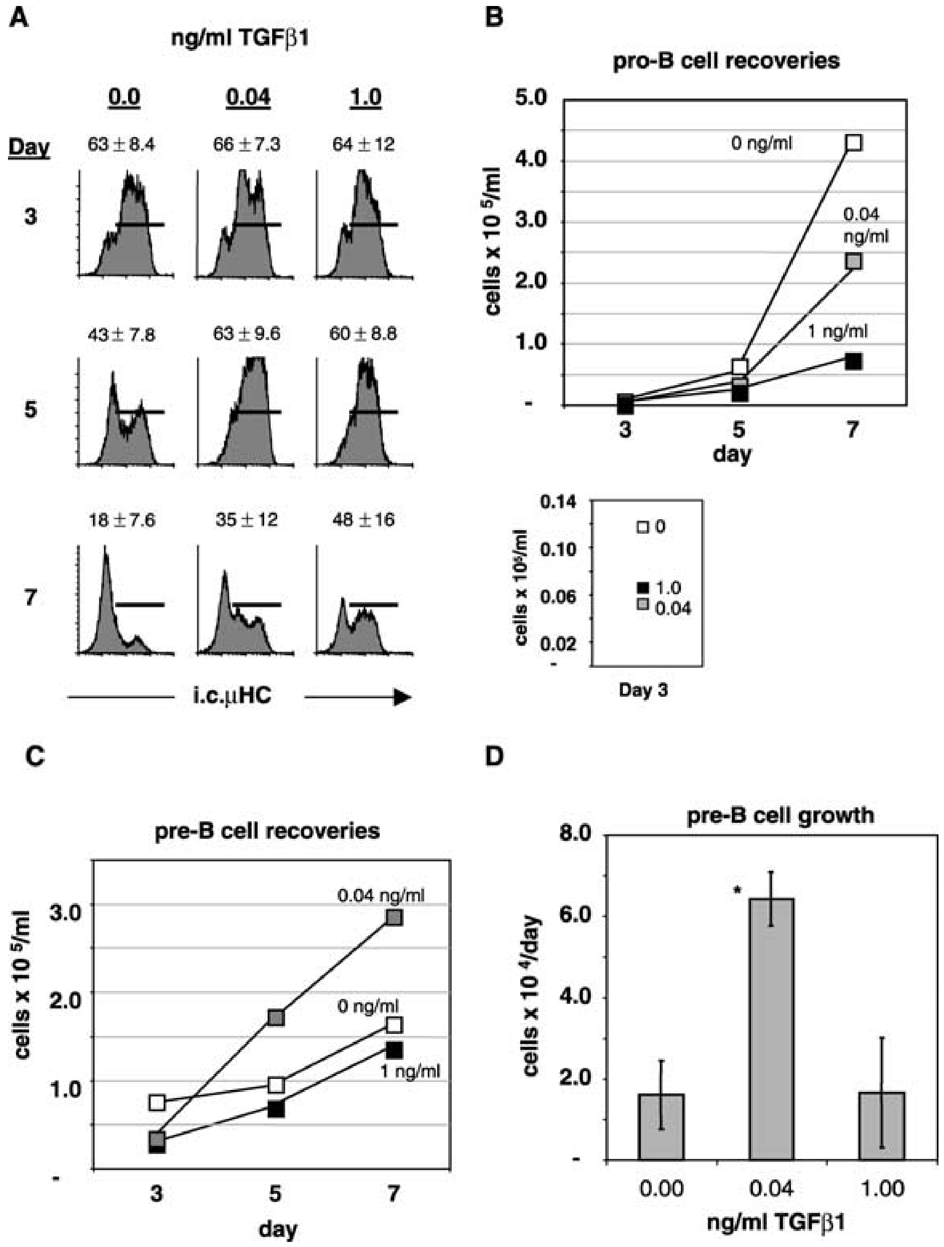

FIGURE 4 Differential effects of TGFβ1 on pro- versus pre-B cells. $B220^+$ BM cells from 4-week-old C57BL/6 mice were treated with IL7 ± the indicated concentrations of TGFβ1. (A) Flow cytometry histograms of i.c.μHC staining within the $B220^+sIgM^-$ BCP gate of cells recovered at the indicated times. Values are the percent i.c.μHC$^+$ within the total viable sample (± standard deviations from 3 experiments). (B) Absolute numbers of pro-B-enriched ($B220^+sIgM^-$i.c.μHC$^-$) cells recovered over time from one representative experiment of three. *Bottom* shows day 3 values on their own scale. (C) Absolute numbers of pre-B ($B220^+sIgM^-$i.c.μHC$^+$) cells over time from the experiment shown in B. (D) Pre-B cell growth is indicated as the average number of cells generated per day. This was calculated as the number of cells at day 7 of culture minus the cell number at day 3 divided by 4 days. The mean ± sample standard deviations from 3 experiments are shown. *$p = 0.0015$ versus 0 ng/ml TGFβ1.

population. By sort-purification of the starting BCP subpopulations, the large BP1$^+$ pre-B cell fraction was identified as containing the cells that are increased in response to TGFβ1.

Reductions in BM-BCP in TGFβ1$^{-/-}$ mice were unanticipated because TGFβ had been previously shown to have inhibitory effects upon the B lineage *in vitro* (Lee *et al.*, 1987; Kincade *et al.*, 1989; Lee *et al.*, 1989; Rehmann and LeBien, 1994; Kee *et al.*, 2001). The reductions *in vivo* may thus be due to an unrecognized necessity for a TGFβ receptor signal directly upon B lineage cells. Alternatively, effects secondary to the TGFβ1 deficiency, e.g. soluble factors produced by infiltrating inflammatory cells, circulating prostaglandins or sex hormones (Kincade *et al.*, 1989; Wang *et al.*, 1995; Kincade *et al.*, 2000), which could be directly regulated by TGFβ1 or induced in response to inflammatory stress may be responsible. Alterations in cellular adhesion may also contribute to dysregulated B lymphopoiesis in the BM (Dittel *et al.*, 1993; Dittel and LeBien, 1995).

B cell development is apparently normal in very young (1.5 week) TGFβ1$^{-/-}$ mice and deteriorates thereafter.

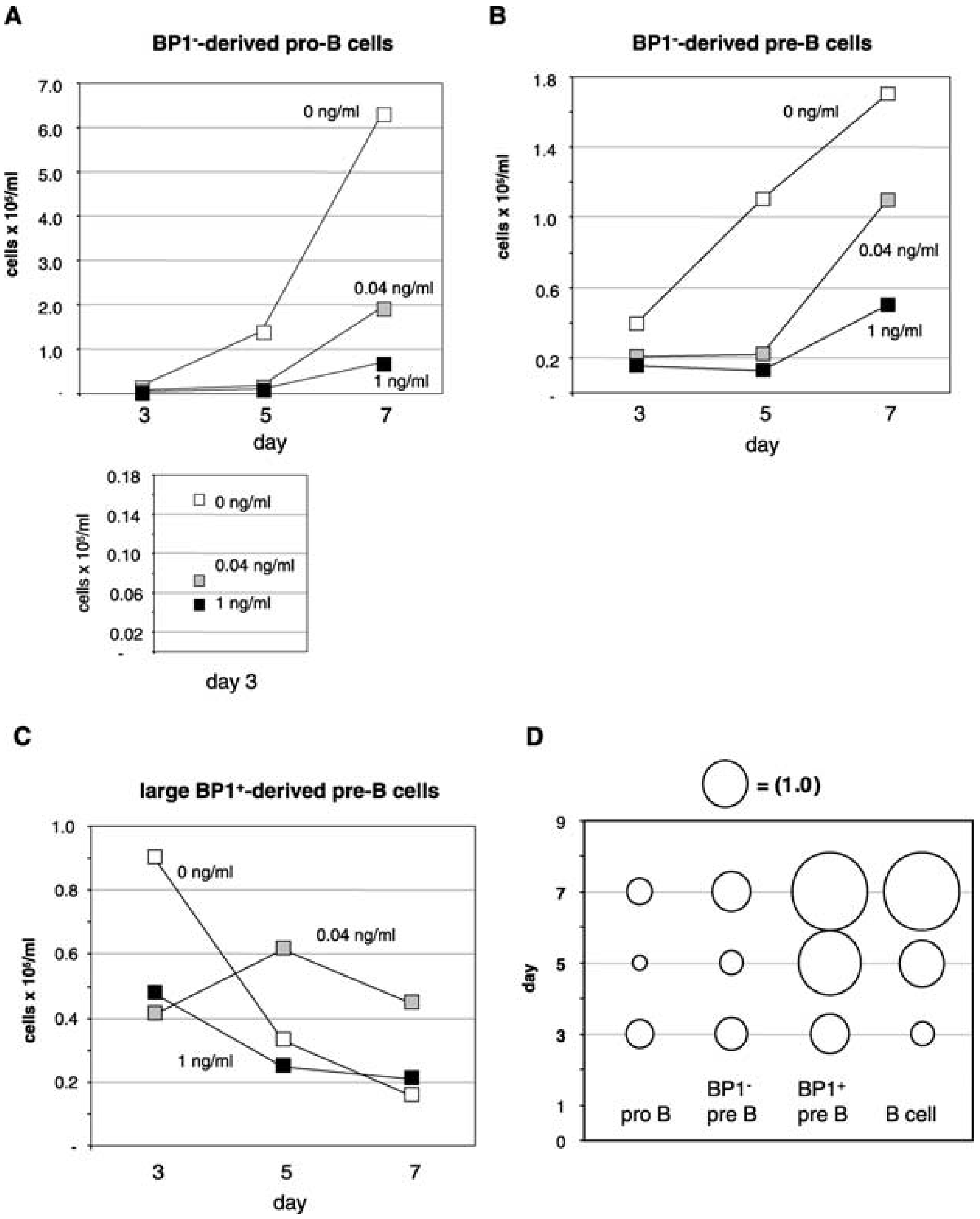

FIGURE 5 Differential sensitivity of BCP subsets to TGFβ1. $CD19^{+}sIgM^{-}$ BCP from C57BL/6 mice were sort-purified into $BP1^{-}$ and large $BP1^{+}$ subsets. The sorted cells were cultured, harvested, and phenotyped as in Fig. 4. (A) Numbers of pro-B cells derived from the $BP1^{-}$ sub-population from a representative experiment. (B) Numbers of pre-B cells derived from the same $BP1^{-}$ sub-population. (C) Pre-B cells derived from the large, $BP1^{+}$ sub-population. Results in A–C are representative of 3 experiments. (D) Summary of relative effects of low-dose TGFβ1 upon B cell development over time in culture. The size of each circle represents the effect of low-dose TGFβ1 in IL7 as a fold change compared to IL7 alone ( = 1.0). Stages of B cell development examined in culture are on the *X* axis, whereas time in culture progresses from bottom-to-top on the *Y* axis.

This age dependency may be due to maternal TGFβ1, acquired *in utero* or during nursing, compensating early in life for the lack of *de novo* production (Letterio *et al.*, 1994) and delaying the onset of defects in B lymphopoiesis. Alternatively, BCP derived from older mice may have a differential sensitivity to TGFβ1 in comparison to those generated earlier in life, as differences have been observed for BCP at different stages of ontogeny (Kearney *et al.*, 1997; Hardy *et al.*, 2000; Igarashi *et al.*, 2001). In either case, the mature B cells found in the BM and periphery of 3- to 5-week old $TGF\beta1^{-/-}$ mice are likely generated at an earlier age when B cell development is unaffected by the TGFβ1 mutation.

$Thy1.2^{+}$ BM cells were examined in $TGF\beta1^{-/-}$ mice because activated T cells have been shown to be responsible for the systemic inflammation that these mice acquire (Kulkarni and Karlsson, 1993; Diebold *et al.*, 1995; Kulkarni *et al.*, 1995; Borkowski *et al.*, 1996; Letterio *et al.*, 1996; McCartney-Francis *et al.*, 1997; Nakabayashi *et al.*, 1997; Kobayashi *et al.*, 1999; McLennan *et al.*, 2000). $Mac1^{+}$ myeloid lineage BM cells were also examined because enhanced myelopoiesis, an apparent consequence of TGFβ1 deficiency (Boivin *et al.*, 1995; Letterio *et al.*, 1996), correlates with suppressed B lymphopoiesis (Buske *et al.*, 2001; Fraker and King, 2001). Macrophages are a component of the cellular infiltrate seen in other tissues, such as the heart, in these mice (Boivin *et al.*, 1995; Kulkarni *et al.*, 1995; Letterio *et al.*, 1996), and their activation products, including interleukin 1 and the interferons, have been shown to be inhibitory for BCP (Dorshkind, 1988; Wang *et al.*, 1995).

The analysis of T and myeloid cells in individual TGFβ1$^{-/-}$ mice indicates variable alterations in the cellular composition of the BM; however, there was no consistent correlation with BCP reductions. Moreover, when TGFβ1$^{-/-}$ mice are rendered deficient in CD8$^{+}$ T cells, by backcrossing to a β2-microglobulin (MHC Class I) deficient genetic background, which prevents the T cell-mediated inflammatory disease, total B220$^{+}$ BM B lineage cells are still reduced (Kobayashi *et al.*, 1999). This deficiency in B lineage cells, as in our studies, is specific to the BM, and is not observed in the spleen. We have attempted to address the role of the T cell-mediated inflammatory response in the BCP deficiency by breeding TGFβ1$^{-/-}$ and TCRα$^{-/-}$ mice. However, no doubly homozygous mutant offspring were obtained.

Another approach has been to examine B cell development in a model where only B lineage cells are unresponsive to TGFβ. In mice conditionally gene-targeted for the TβRII subunit of the TGFβ receptor specifically in B cells, the early B lineage cells in the BM were reportedly unchanged in comparison to the controls (Cazac and Roës, 2000). However, the CD19*cre* deletion system used in these studies is less efficient in late pre-B cells (75–80%) than in splenic B cells (90–95%) (Rickert *et al.*, 1997). It is thus unclear whether and at what efficiency TβRII deletion occurs at earlier stages of B cell development, since TGFβ responsiveness was not examined in the BM B lineage cells of these mice. However, in the periphery, where B lineage cells had defective TGFβ receptor signaling, populations of mature B lymphocytes were increased, as were serum levels of anti-nuclear antibodies indicating a direct inhibitory role for TGFβ on B lineage cells at later stages (Cazac and Roës, 2000).

If BCP deficiencies in TGFβ1$^{-/-}$ mice are due to a requirement for a direct TGFβ receptor signal on these cells, we reasoned that using a defined culture system with rIL7 and purified BM B lineage cells from normal mice, we could identify sub-populations that benefit from exposure to exogenous TGFβ1. In IL7-containing cultures, pro-B cell recoveries were consistently decreased by low-dose TGFβ1 treatment. An often-overlooked population of i.c. μHC$^{+}$ pre-B cells, included in Hardy's Fraction B, is also reduced by TGFβ1 in culture. By contrast, the numbers of pre-B cells derived from large BP1$^{+}$ BCP were increased. Notably, the more severe *in vivo* BCP reductions in TGFβ1$^{-/-}$ mice begin in Fraction C, which corresponds to the large BP1$^{+}$ pre-B cells in our cultures.

The effects of TGFβ on cell cycle and apoptosis may provide an alternative explanation for our *in vitro* data. Pro-B cells proliferating in response to IL-7 eventually mature into i.c. μHC$^{+}$ pre-B cells and then exit cell cycle. At low doses, TGFβ may inhibit proliferation of the pro-B cells, which then may complete IgH chain gene rearrangement and express μHC. This outcome would result in the observed decrease in pro-B cells and increase in pre-B cells. Higher doses of TGFβ may induce pro-B cell apoptosis, thus accounting for the decrease in both pro- and pre-B cells. However, this interpretation does not account for the net increase in pre-B cells with low dose TGF-β in cultures initiated with large BP1$^{+}$ cells, 90–99% of which already express μHC.

In conclusion, TGFβ1 influences B cell development in multiple ways including directly inhibiting pro-B cell/early pre-B cell populations. The BP1$^{+}$ pre-B cells are unique among B lineage cells in their positive response to exogenous TGFβ1. It is currently unclear why the large pre-B cells respond to TGFβ in this way. It has been suggested that the stability of the preBCR, which varies depending upon the μHC variable region, may regulate cellular viability and proliferation (Melchers *et al.*, 2000). A pre-B cell with a well-fitting preBCR would receive proliferative/viability signals *via* the receptor itself, and consequently the cell could be changed in other ways to give it the greatest advantage over cells with a poorly fitting preBCR. Differential responsiveness to TGFβ may be one such phenotypic change and would be advantageous for expanding the numbers of these positively selected pre-B cells.

**_Acknowledgements_**

We thank Max D. Cooper, John F. Kearney, Flavius Martin and Robert P. Stephan for discussion and assistance, Larry Gartland for cell sorting, Jeff Nyswaner for animal care, and Ann Brookshire for editorial assistance. Supported by National Institutes of Health Grants AI48098 and HD36312; D.A. Kaminski was supported by T32AI07051-26.

**_References_**

Boivin, G.P., O'Toole, B.A., Orsmby, I.E., Diebold, R.J., Eis, M.J., Doetschman, T. and Kier, A.B. (1995) "Onset and progression of pathological lesions in transforming growth factor-β 1-deficient mice", *American Journal of Pathology* **146**, 276–288.

Bonyadi, M., Rusholme, S.A., Cousins, F.M., Su, H.C., Biron, C.A., Farrall, M. and Akhurst, R.J. (1997) "Mapping of a major genetic modifier of embryonic lethality in TGF β 1 knockout mice", *Nature Genetics* **15**, 207–211.

Borghesi, L.A., Smithson, G. and Kincade, P.W. (1997) "Stromal cell modulation of negative regulatory signals that influence apoptosis and proliferation of B lineage lymphocytes", *The Journal of Immunology* **159**, 4171–4179.

Borkowski, T.A., Letterio, J.J., Farr, A.G. and Udey, M.C. (1996) "A role for endogenous transforming growth factor β1 in Langerhans cell biology: the skin of transforming growth factor β1 null mice is devoid of epidermal Langerhans cells", *Journal of Experimental Medicine* **184**, 2417–2422.

Bottinger, E.P., Letterio, J.J. and Roberts, A.B. (1997) "Biology of TGF-β in knockout and transgenic mouse models", *Kidney International* **51**, 1355–1360.

Buske, C., Feuring-Buske, M., Antonchuk, J., Rosten, P., Hogge, D.E., Eaves, C.J. and Humphries, R.K. (2001) "Overexpression of HOXA10 perturbs human lymphomyelopoiesis *in vitro* and *in vivo*", *Blood* **97**, 2286–2292.

Candeias, S., Muegge, K. and Durum, S.K. (1997) "IL-7 receptor and VDJ recombination: trophic versus mechanistic actions", *Immunity* **6**, 501–508.

Cazac, B.B. and Roës, J. (2000) "TGF-β receptor controls B cell responsiveness and induction of IgA *in vivo*", *Immunity* **13**, 443–451.

Christ, M., McCartney-Francis, N.L., Kulkarni, A.B. and Ward, J.M. (1994) "Immune dysregulation in TGF-β1-deficient mice", *Journal of Immunology* **153**, 1936–1946.

Corcoran, A.E., Riddell, A., Krooshoop, D. and Venkitaraman, A.R. (1998) "Impaired immunoglobulin gene rearrangement in mice lacking the IL-7 receptor", *Nature* **391**, 904–907.

Dang, H., Geiser, A.G., Letterio, J.J., Nakabayashi, T., Kong, L., Fernandes, G. and Talal, N. (1995) "SLE-like autoantibodies and Sjogren's syndrome-like lymphoproliferation in TGF-β knockout mice", *Journal of Immunology* **155**, 3205–3212.

Diebold, R.J., Eis, M.J., Yin, M., Ormsby, I., Boivin, G.P., Darrow, R.J., Safitz, J.E. and Doetchman, T. (1995) "Early-onset multifocal inflammation in the transforming growth factor β1-null mouse is lymphocyte-mediated", *Proceedings of the National Academy of Sciences of the United States of America* **92**, 12215–12219.

Dickson, M.C., Martin, J.S., Cousins, F.M., Kulkarni, A.B., Karlsson, S. and Akhurst, R.J. (1995) "Defective haematopoiesis and vasculogenesis in transforming growth factor-β1 knock out mice", *Development* **121**, 1845–1854.

Dittel, B.N. and LeBien, T.W. (1995) "Reduced expression of vascular cell adhesion molecule-1 on bone marrow stromal cells isolated from marrow transplant recipients correlates with a reduced capacity to support human B lymphopoiesis *in vitro*", *Blood* **86**, 2833–2841.

Dittel, B.N., McCarthy, J.B., Wayner, E.A. and LeBien, T.W. (1993) "Regulation of human B-cell precursor adhesion to bone marrow stromal cells by cytokines that exert opposing effects on the expression of vascular cell adhesion molecule-1 (VCAM-1)", *Blood* **81**, 2272–2282.

Dorshkind, K. (1988) "IL-1 inhibits B cell differentiation in long term bone marrow cultures", *Journal of Immunology* **141**, 531–538.

Fraker, P.J. and L.E. (2001) "A distinct role for apoptosis in the changes in lymphopoiesis and myelopoiesis created by deficiencies in zinc", *FASEB Journal* **15**, 2572–2578.

Gimble, J.M., Pietrangeli, C., Henley, A., Dorheim, M.A., Silver, J., Namen, A., Takeichi, M., Goridis, C. and Kincade, P.W. (1989) "Characterization of murine bone marrow and spleen-derived stromal cells: analysis of leukocyte marker and growth factor mRNA transcript levels", *Blood* **74**, 303–311.

van Ginkel, F.W., Wahl, S.W., Kearney, J.F., Kweon, M.N., Fujihashi, K., Burrows, P.D., Kiyono, H. and McGhee, J.R. (1999) "Parital IgA deficiency with increased Th2-type cytokines in TGF-β1 knockout mice", *The Journal of Immunology* **163**, 1951–1957.

Hardy, R.R. (1992) "Variable gene usage, physiology and development of Ly-$1^+$ ($CD5^+$) B cells", *Current Opinion in Immunology* **4**, 181–185.

Hardy, R.R., Carmack, C.E., Shinton, S.A., Kemp, J.D. and Hayakawa, K. (1991) "Resolution and characterization of pro-B and pre-pro-B cell stages in normal mouse bone marrow", *Journal of Experimental Medicine* **173**, 1213–1225.

Hardy, R.R., Wasserman R., Li, Y.S., Shinton, S.A., Hayakawa, K. (2000) "Response by B cell precursors to pre-B receptor assembly: differences between fetal liver and bone marrows." *Current Topics in Microbiology and Immunology* **252**, 25–30.

Hocevar, B.A. and Howe, P.H. (1998) "Mechanisms of TGF-β-induced cell cycle arrest", *Mineral and Electrolyte Metabolism* **24**, 131–135.

Igarashi, H., Kouro, T., Yokota, T., Comp, P.C. and Kincade, P.W. (2001) "Age and stage dependency of estrogen receptor expression by lymphocyte precursors", *Proceedings of the National Academy of Sciences of the United States of America* **98**, 15131.

Itoh, N., Yasunaga, M., Hirashaima, M., Yoshida, O. and Nishikawa, S.I. (1996) "Role of IL-7 and KL in activating molecules controlling the G1/S transition of B precursor cells", *International Immunology* **8**, 317–323.

Kaartinen, V., Voncken, J.W., Shuler, C., Warburton, D., Bu, D., Heisterkamp, N. and Groffen, J. (1995) "Abnormal lung development and cleft palate in mice lacking TGF-β3 indicates defects of epithelial-mesenchymal interaction", *Nature Genetics* **11**, 415–421.

Kearney, J.F., Won, W.J., Benedict, C., Moratz, C., Zimmer, P., Oliver, A., Martin, F. and Shu, F. (1997) "B cell development in mice", *International Reviews of Immunology* **15**, 207–241.

Kee, B.L., Rivera, R.R. and Murre, C. (2001) "Id3 inhibits B lymphocyte progenitor growth and survival in response to TGFβ", *Nature Immunology* **2**, 242–247.

Kim, P.H. and Kagnoff, M.F. (1990) "Transforming growth factor β1 increases IgA isotype switching at the clonal level", *Journal of Immunology* **145**, 3773–3778.

Kim, L.T. and Yamada, K.M. (1997) "The regulation of expression of integrin receptors", *Proceedings of the Society for Experimental Biology and Medicine* **214**, 123–131.

Kincade, P.W., Lee, G., Pietrangeli, C.E., Hayashi, S. and Gimble, J.M. (1989) "Cells and molecules that regulate B lymphopoiesis in bone marrow", *Annual Review of Immunology* **7**, 111–143.

Kincade, P.W., Medina, K.L., Payne, K.J., Rossi, M.I., Tudor, K.S., Yamashita, Y. and Kouro, T. (2000) "Early B-lymphocyte precursors and their regulation by sex steroids", *Immunological Reviews* **175**, 128–137.

King, A.G., Wierda, D. and Landreth, K.S. (1988) "Bone marrow stromal cell regulation of B-lymphopoiesis. I. The role of macrophages, IL-1, and IL-4 in pre-B cell maturation", *Journal of Immunology* **141**, 2016–2026.

Kobayashi, S., Yoshida, K., Ward, J.M., Letterio, J.J., Longenecker, G., Yaswen, L., Mittleman, B., Mozes, E., Roberts, A.B., Karlsson, S. and Kulkarni, A.B. (1999) "β2-microglobulin-deficient background ameliorates lethal phenotype of the TGF-β 1 null mouse", *Journal of Immunology* **163**, 4013–4019.

Kulkarni, A.B. and Karlsson, S. (1993) "Transforming growth factor-β1 knockout mice. A mutation in one cytokine gene causes a dramatic inflammatory disease", *American Journal of Pathology* **143**, 3–9.

Kulkarni, A.B., Huh, C.G., Becker, D., Geiser, A., Lyght, M., Flanders, K.C., Roberts, A.B., Sporn, M.B., Ward, J.M. and Karlsson, S. (1993) "Transforming growth factor β1 null mutation in mice causes excessive inflammatory response and early death", *Proceedings of the National Academy of Sciences of the United States of America* **90**, 770–774.

Kulkarni, A.B., Ward, J.M., Yaswen, L., Mackall, C.L., Bauer, S.R., Huh, C.G., Gress, R.E. and Karlsson, S. (1995) "Transforming growth factor-β1 null mice. An animal model for inflammatory disorders", *American Journal of Pathology* **146**, 264–275.

Larsson, J., Goumans, M.J., Sjostrand, L.J., van Rooijen, M.A., Ward, D., Leveen, P., Xu, X., ten Dijke, P., Mummery, C.L. and Karlsson, S. (2001) "Abnormal angiogenesis but intact hematopoietic potential in TGF-β type I receptor-deficient mice", *EMBO Journal* **20**, 1663–1673.

Lebman, D.A., Lee, F.D. and Coffman, R.L. (1990) "Mechanism for transforming growth factor β and IL-2 enhancement of IgA expression in lipopolysaccharide-stimulated B cell cultures", *Journal of Immunology* **144**, 952–959.

Lee, G., Ellingsworth, L.R., Gillis, S., Wall, R. and Kincade, P.W. (1987) "β transforming growth factors are potential regulators of B lymphopoiesis", *Journal of Experimental Medicine* **166**, 1290–1299.

Lee, G., Namen, A.E., Gillis, S., Ellingsworth, L.R. and Kincade, P.W. (1989) "Normal B cell precursors responsive to recombinant murine IL-7 and inhibition of IL-7 activity by transforming growth factor-β", *Journal of Immunology* **142**, 3875–3883.

Letterio, J.J. and Roberts, A.B. (1998) "Regulation of immune responses by TGF-β", *Annual Review of Immunology* **16**, 137–161.

Letterio, J.J., Geiser, A.G., Kulkarni, A.B., Roche, N.S., Sporn, M.B. and Roberts, A.B. (1994) "Maternal rescue of transforming growth factor-β1 null mice", *Science* **264**, 1936–1938.

Letterio, J.J., Geiser, A.G., Kulkarni, A.B., Dang, H., Kong, L., Nakabayashi, T., Mackall, C.L., Gress, R.E. and Roberts, A.B. (1996) "Autoimmunity associated with TGF-β1 deficiency in mice is dependent on MHC Class II antigen expression", *The Journal of Clinical Investigation* **98**, 2109–2119.

Li, Y.S., Hayakawa, K. and Hardy, R.R. (1993) "The regulated expression of B lineage associated genes during B cell differentiation in bone marrow and fetal liver", *Journal of Experimental Medicine* **178**, 951–960.

Li, Y.S., Wasserman, R., Hayakawa, K. and Hardy, R.R. (1996) "Identification of the earliest B lineage stage in mouse bone marrow", *Immunity* **5**, 527–535.

Marshall, L.J., Fleming, H.E., Wu, G.E. and Paige, C.J. (1998) "Modulation of the IL-7 dose-response threshold during pro B cell differentiation is dependent on pre B cell receptor expression", *The Journal of Immunology* **161**, 6038–6045.

Martin, J.S., Dickson, M.C., Cousins, F.M., Kulkarni, A.B., Karlsson, S. and Akhurst, R.J. (1995) "Analysis of homozygous TGF β1 null mouse embryos demonstrates defects in yolk sac vasculogenesis and hematopoiesis", *Annals of the New York Academy of Sciences* **752**, 300–308.

Massague, J., Cheifetz, S., Laiho, M., Ralph, D.A., Weis, F.M. and Zentella, A. (1992) "Transforming growth factor-β", *Cancer Surveys* **12**, 81–103.

McCartney-Francis, N.L. and Wahl, S.M. (1994) "Transforming growth factor β: a matter of life and death", *Journal of Leukocyte Biology* **55**, 401–409.

McCartney-Francis, N.L., Mizel, D.E., Frazier-Jessen, M., Kulkarni, A.B., McCarthy, J.B. and Wahl, S.M. (1997) "Lacrimal gland inflammation is responsible for ocular pathology in TGF-β1 null mice", *American Journal of Pathology* **151**, 1281–1288.

McLennan, I.S., Poussart, Y. and Koishi, K. (2000) "Development of skeletal muscles in transforming growth factor-β1 (TGF-β1) null-mutant mice", *Developmental Dynamics* **217**, 250–256.

Meffre, E., Casellas, R. and Nussenzweig, M.C. (2000) "Antibody regulation of B cell development", *Nature Immunology* **1**, 379–385.

Melchers, F., ten Boekel, E., Seidl, T., Kong, X.C., Yamagami, T., Onishi, K., Shimizu, T., Rolink, A.G. and Andersson, J. (2000) "Repertoire selection by pre-B-cell receptors and B-cell receptors, and genetic control of B-cell development from immature to mature B cells", *Immunological Reviews* **175**, 33–46.

Nakabayashi, T., Letterio, J.J., Geiser, A.G., Kong, L., Ogawa, N., Zhao, W., Koike, T., Fernandes, G., Dang, H. and Talal, N. (1997) "Up-regulation of cytokine mRNA, adhesion molecule proteins, and MHC class II proteins in salivary glands of TGF-β1 knockout mice: MHC class II is a factor in the pathogenesis of TGF-β1 knockout mice", *Journal of Immunology* **158**, 5527–5535.

Olsen, N.J., Gu, X. and Kovacs, W.J. (2001) "Bone marrow stromal cells mediate androgenic suppression of B lymphocyte development", *Journal of Clinical Investigation* **108**, 1697–1704.

Osmond, D.G. (1991) "Proliferation kinetics and the lifespan of B cells in central and peripheral lymphoid organs", *Current Opinion in Immunology* **3**, 179–185.

Papavasiliou, F., Jankovic, M., Gong, S. and Nussenzweig, M.C. (1997) "Control of immunoglobulin gene rearrangements in developing B cells", *Current Opinion in Immunology* **9**, 233–238.

Peschon, J.J., Morrissey, P.J., Grabstein, K.H., Ramsdell, F.J., Maraskovsky, E., Gliniak, B.C., Park, L.S., Ziegler, S.F., Williams, D.E., Ware, C.B., *et al.*, (1994) "Early lymphocyte expansion is severely impaired in interleukin 7 receptor-deficient mice", *Journal of Experimental Medicine* **180**, 1955–1960.

Ravitz, M.J. and Wenner, C.E. (1997) "Cyclin-dependent kinase regulation during G1 phase and cell cycle regulation by TGF-β", *Advances in Cancer Research* **71**, 165–207.

Rehmann, J.A. and LeBien, T.W. (1994) "Transforming growth factor-β regulates normal human pre-B cell differentiation", *International Immunology* **6**, 315–322.

Rickert, R.C., Röes, J. and Rajewsky, K. (1997) "B lymphocyte-specific, Cre-mediated mutagenesis in mice", *Nucleic Acids Research* **25**, 1317–1318.

Rifkin, D.B., Kojima, S., Abe, M. and Harpel, J.G. (1993) "TGF-β: structure, function, and formation", *Thrombosis and Haemostasis* **70**, 177–179.

Roberts, A.B., McCune, B.K. and Sporn, M.B. (1992) "TGF-β: regulation of extracellular matrix", *Kidney International* **41**, 557–559.

Robledo, M.M., Sanz-Rodriguez, F., Hidalgo, A. and Teixido, J. (1998) "Differential use of very late antigen-4 and -5 integrins by hematopoietic precursors and myeloma cells to adhere to transforming growth factor-β1-treated bone marrow stroma", *Journal of Biological Chemistry* **273**, 12056–12060.

Rolink, A.G., ten Boekel, E., Yamagami, T., Ceredig, R., Andersson, J. and Melchers, F. (1999) "B cell development in the mouse from early progenitors to mature B cells", *Immunology Letters* **68**, 89–93.

Sanford, L.P., Ormsby, I., Gittenbergerdegroot, A.C., Sariola, H., Friedman, R., Boivin, G.P., Cardell, E.L. and Doetschman, T. (1997) "TGF-β2 knockout mice have multiple developmental defects that are non-overlapping with other TGF-β knockout phenotypes", *Development* **124**, 2659–2670.

Shockett, P. and Stavnezer, J. (1991) "Effect of cytokines on switching to IgA and alpha germline transcripts in the B lymphoma I.29 mu. Transforming growth factor-β activates transcription of the unrearranged C alpha gene", *Journal of Immunology* **147**, 4374–4383.

Shull, M.M., Ormsby, I., Kier, A.B., Pawlowski, S, Diebold, R.J., Yin, M. and Allen, R. (1992) "Targeted disruption of the mouse transforming growth factor-β1 gene results in multifocal inflammatory disease", *Nature* **359**, 693–699.

Tudor, K.S., Payne, K.J., Yamashita, Y. and Kincade, P.W. (2000) "Functional assessment of precursors from murine bone marrow suggests a sequence of early B lineage differentiation events", *Immunity* **12**, 335–345.

Wahl, S.M. (1994) "Transforming growth factor β: the good, the bad, and the ugly", *Journal of Experimental Medicine* **180**, 1587–1590.

Wang, J., Lin, Q., Langston, H. and Cooper, M.D. (1995) "Resident bone marrow macrophages produce type 1 interferons that can selectively inhibit interleukin-7-driven growth of B lineage cells", *Immunity* **3**, 475–484.

Wasserman, R., Li, Y.S. and Hardy, R.R. (1997) "Down-regulation of terminal deoxynucleotidyl transferase by Ig heavy chain in B lineage cells", *Journal of Immunology* **158**, 1133–1138.

Yaswen, L., Kulkarni, A.B., Fredrickson, T., Mittleman, B., Schiffman, R., Payne, S., Longenecker, G., Mozes, E. and Karlsson, S. (1996) "Autoimmune manifestations in the transforming growth factor-β1 knockout mouse", *Blood* **87**, 1439–1445.

